# Antiphospholipid Syndrome: Multiple Manifestations in a Single Patient—A High Suspicion Is Still Needed

**DOI:** 10.1155/2017/5797041

**Published:** 2017-05-23

**Authors:** Uroosa Ibrahim, Shiksha Kedia, Gwenalyn Garcia, Jean Paul Atallah

**Affiliations:** Department of Hematology/Oncology, Staten Island University Hospital, 475 Seaview Avenue, Staten Island, NY 10305, USA

## Abstract

Antiphospholipid Syndrome (APS) is an autoimmune disorder with clinical and laboratory features of vascular thrombosis, pregnancy loss, and persistent antiphospholipid antibodies (aPLs). The pathophysiology is thought to involve the activation of endothelial cells, monocytes, platelets, and complement by aPLs. Disease can range from asymptomatic to rapidly fatal catastrophic APS. We present a case of a 34-year-old male referred for pancytopenia and splenomegaly. On examination, he had decreased sensation and 4/5 power in the left upper extremity. A lacy, purplish rash was noted on the trunk and upper extremity. MRI of brain showed acute/subacute lacunar infarctions. Laboratory studies revealed an elevated lactate dehydrogenase level, bilirubin and ferritin, decreased haptoglobin, and positive Coombs test. Antinuclear antibody test was negative and antiphospholipid antibody panel revealed positivity for anti-cardiolipin IgG and IgM, antiphosphatidylserine IgG, and anti-*β*2-glycoprotein IgG. The patient was diagnosed with primary APS. Pancytopenia is relatively rare in primary APS and is more often seen in secondary APS. Our patient demonstrated involvement of multiple organ systems as well as livedo reticularis and autoimmune-related findings such as Raynaud phenomenon and Coombs positive hemolytic anemia. We discuss the various clinical and laboratory findings in patients with APS that aid in diagnosis, as well as important management considerations.

## 1. Introduction

Antiphospholipid Syndrome (APS) is a potentially catastrophic syndrome that can lead to significant morbidity if not appropriately recognized and treated. The disease can involve multiple organ system and manifest various hematologic abnormalities. Pancytopenia and autoimmune hemolytic anemia are unusual presentations of the disease that were seen in our patient.

## 2. Case

A 34-year-old male was referred to the hematology clinic for pancytopenia and splenomegaly, initially discovered on workup for gradual onset left arm numbness and weakness of a few days' duration. Complete blood count showed hemoglobin of 8.8 g/dL, MCV of 95 fL, white blood cell count of 3,300/*μ*L, and platelet count of 92,000/*μ*L. His past medical history was significant for seizure disorder and Raynaud phenomenon. Medications included methadone and quetiapine. Family history was significant for myasthenia gravis in his mother. Social history was significant for a history of cocaine and heroin abuse, last used two weeks prior to presentation.

**Figure 1:**
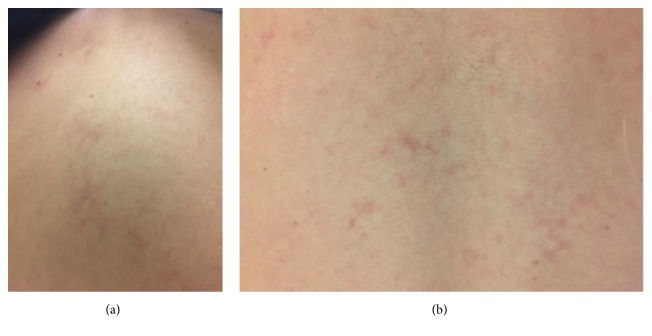
Lacy, purplish rash on the upper and lower back.

On examination, pallor and mild icterus were noted. The spleen tip was palpable two finger breadths below the costal margin. No peripheral lymphadenopathy was noted. Neurologic exam showed decreased sensation and 4/5 power in the left upper extremity. A lacy, purplish rash was noted on the trunk and upper extremity (Figure 1).

Initial workup showed an elevated total bilirubin of 3.3 mg/dL with otherwise normal liver function. LDH was elevated at 273 U/L and low haptoglobin levels of <20 mg/dL, suggesting hemolysis. Creatinine was measured at 1.3 mg/dL with a GFR of 70 mL/min. Urinalysis showed moderate blood and trace protein. Direct Coombs was positive for IgG and C3. Flow cytometry was negative for paroxysmal nocturnal hemoglobinuria. ESR was elevated at 68. Ferritin was modestly elevated at 334. The rest of the workup, including vitamin B12, folate, serum protein electrophoresis, serum immunofixation, and quantitative immunoglobulins, were normal. Antinuclear antibodies (ANA), anti-ds DNA, and anti-smooth muscle antibodies were negative as well.

Bone marrow studies were negative. CT abdomen and pelvis revealed splenomegaly of 17.6 cm. Otherwise, there was no evidence of lymphadenopathy. MRI of brain showed multiple lacunar infarctions involving the bilateral posterior parietal lobes. There was a chronic lacunar infarct in the right frontal lobe deep white matter. There were also areas of subcortical signal abnormality and volume loss in the bilateral parietal lobes/postcentral gyri most compatible with small chronic infarctions but which can also be seen in demyelinating disorders. Subsequent lumbar puncture was negative for oligoclonal bands.

Antiphospholipid antibody panel revealed positivity for anti-cardiolipin IgG at 124 GPL and IgM at 17 MPL, anti-phosphatidylserine IgG at >100 U, and anti-*β*2-glycoprotein IgG at 95 U/mL. Anti-phosphatidylserine IgM and anti-*β*2-glycoprotein IgG were negative. PTT was slightly elevated at 40 sec and PT was normal. Complement 3 and 4 were minimally depressed at 73 mg/dL and 9 mg/dL, respectively.

Given the positive antiphospholipid antibody panel and cerebral infarctions in the absence of an underlying connective tissue disorder, the patient was diagnosed with primary antiphospholipid antibody syndrome (APS). He was started on 81 mg of aspirin and 1 mg/kg of prednisone daily. Hemoglobin, white blood cell count, and platelet count have improved on several weeks of tapering steroid therapy. Repeat antibody testing was not done because the patient was lost to follow-up.

## 3. Discussion

APS is an autoimmune disorder that encompasses clinical and laboratory features of vascular thrombosis, pregnancy loss, and persistent antiphospholipid antibodies (aPLs) [1, 2]. The pathophysiology of APS, though unclear, is thought to involve the activation of endothelial cells, monocytes, platelets, and complement by aPLs, resulting in a prothrombotic state [3].

Clinical manifestations of APS can be diverse with involvement of multiple organ systems. Disease can range from asymptomatic to rapidly fatal catastrophic APS. Deep venous thrombosis is the most common event, occurring in nearly 40% of patients [4]. In addition, thrombotic events at unusual sites such as the cerebral venous sinuses, renal and adrenal veins, hepatic veins, and retinal veins may occur, resulting in organ damage. Arterial ischemic events include cerebrovascular accidents, acute coronary syndrome, and amaurosis fugax [3]. Thrombosis in the microvasculature may manifest clinically in the form of cutaneous lesions (most commonly livedo reticularis), limb or visceral gangrene, acute renal insufficiency, valvular heart disease, or alveolar hemorrhage [5, 6]. Early and late fetal losses have been shown to occur in over 33% and 15% of pregnancies, respectively [4].

Thrombocytopenia and hemolytic anemia are hematological manifestations. Isolated thrombocytopenia is the most common hematologic abnormality in APS, occurring in about 30% of patients, and is multifactorial in etiology [4, 7]. Immune-mediated platelet destruction, consumption related to thrombotic microangiopathy, and hypersplenism from portal vein thrombosis have been postulated to contribute to the thrombocytopenia. Rare cases of bone marrow necrosis and hemophagocytic syndrome in the setting of APS have been reported [7]. Alterations in bone marrow microcirculation can lead to ischemia and subsequent necrosis. The pathogenesis of BMN associated with APS is also unclear [8]. Autoimmune hemolytic anemia (AIHA) has been reported to occur in about 10% of patients [4]. About 5–10% of patients have both thrombocytopenia and AIHA, otherwise known as Evans syndrome [9, 10]. However, pancytopenia is relatively rare in primary APS and is more often seen in secondary APS [4].

Our patient demonstrated involvement of multiple organ systems including central nervous system resulting in a cerebrovascular accident, renal involvement suggested by decreased creatinine and presence of blood and protein in the urine, livedo reticularis as a cutaneous manifestation, and autoimmune-related findings such as Raynaud's phenomenon and Coombs positive hemolytic anemia. His hematologic picture was that of pancytopenia. We postulate that the thrombocytopenia and leukopenia may have had an immune-mediated etiology as well, given that all three hematologic indices improved after treatment with steroids. Normal bone marrow studies excluded bone marrow necrosis or other underlying primary marrow disorders.

Livedo reticularis, a lace like purplish cutaneous lesion, is a hallmark dermatologic manifestation of APS. It is due to superficial venous thrombosis and thrombophlebitis [11]. It occurs in 13% of patients with APSs [12]. The presence of livedo reticularis has been reported to be associated with an increased risk of arterial events [6]. Skin nodules, leg ulcers, and peripheral skin gangrene have been reported [11, 13].

Traditionally, APS has been divided into primary and secondary forms depending on the presence or absence of a connective tissue disorder, most commonly systemic lupus erythematosus (SLE) [10, 14]. One large study found the two entities to have a similar clinical course, except for an increased incidence of arthritis, livedo reticularis, low complements, thrombocytopenia, AIHA, and leukopenia in secondary APS [4]. However, presence of antibodies to nuclear antigen, double stranded DNA suggests secondary APS which were absent in our case. On the contrary, low complement levels were noted.

In terms of our patient's neurological manifestations, use of heroin and cocaine are confounding factors. Cocaine is known to cause vasospasm of the cerebral arteries resulting in ischemic strokes, as well as a thrombotic vasculopathy with microthrombotic disease leading to cerebral infarctions [15, 16]. Levamisole in adulterated cocaine can cause autoimmunity resulting in a high titer of autoantibodies including anti-neutrophil cytoplasmic antibodies (ANCA), ANA, and antiphospholipid antibodies [17]. Cocaine may also have a delayed effect in triggering a prothrombotic state because of endothelial activation which may act synergistically with antiphospholipid antibodies to cause a prothrombotic event, such as in our patient [18].

Thromboprophylaxis is the mainstay of the treatment of APS. Anticoagulation with warfarin is the standard of care for patients who have had a prior thrombotic event. Long-term anticoagulation is recommended for patients with a definite diagnosis of APS given the high risk of thrombosis. Use of agents that may cause vasospasm, such as cocaine, is strongly discouraged since it can exacerbate the underlying thrombotic state leading to clinical thrombosis. In patients without a prior thrombotic event, low-dose aspirin may be considered for patients with primary APS at high risk for thrombosis. For patients with APS lesions on MRI that are consistent with the APS, low-dose aspirin therapy is a reasonable initial approach. Warfarin therapy may be appropriate in patients who, while on aspirin, develop documented cognitive deficits or clear progression of the white matter lesions on serial MRI [19]. Hydroxychloroquine plus low-dose aspirin is recommended for patients with APS and associated SLE. Control of other cardiovascular risk factors is recommended for all patients [20]. Severe or symptomatic thrombocytopenia and AIHA are treated with steroids in the first-line setting [21].

## 4. Conclusion

Our case illustrates the importance of including autoimmune disorders like APS in the differential diagnosis of pancytopenia/AIHA, particularly in the setting of a suggestive clinical history and physical findings. Moreover, this case is of interest due to multiple manifestations of APS in a single patient.
